# Early Serum Procalcitonin Level after Primary Total Hip Replacement

**DOI:** 10.1155/2013/927636

**Published:** 2013-05-13

**Authors:** Samy Bouaicha, Samuel Blatter, Beat K. Moor, Katharina Spanaus, Claudio Dora, Clément M. L. Werner

**Affiliations:** ^1^Department of Orthopaedics, Balgrist University Hospital Zurich, Zurich, Switzerland; ^2^Division of Traumatology, University Hospital Zurich, Zurich, Switzerland; ^3^Institute for Clinical Chemistry, University Hospital, Zurich, Switzerland

## Abstract

*Background*. Procalcitonin (PCT) is a useful surrogate marker for the differentiation of postoperative infection and unspecific inflammatory reaction after surgery. It is known that postoperative course of the PCT serum level varies with type of surgery. No data exists about the postoperative course of serum PCT levels after primary total hip replacement (THR). *Purpose*. To characterize early postoperative serum PCT levels in uneventful primary THR compared to postoperative levels of different frequently used inflammatory blood parameters. *Method*. We prospectively investigated 31 patients. Blood samples were taken preoperatively and for 5 days postoperatively. PCT levels were compared with C-reactive protein (CRP), interleukin-6 (IL-6), and blood leucocyte counts (WBC). *Results*. In uneventful THR PCT levels showed a uniform low-level course with a peak at the second postoperative day. At the fifth day values returned to almost preoperative levels. On contrary, CRP levels remained high during the entire observational period. Only IL-6 levels showed a peak at postoperative day one with a quick and uniform return to preoperative levels. *Conclusion*. Similar to observations in cardiothoracic, intestinal, and neural surgeries, postoperative course of PCT after primary THR showed a uniform low-level course with a peak at the second postoperative day but below expected levels in systemic infections.

## 1. Introduction

Postoperative elevation of proinflammatory cytokines due to surgical trauma and healing process is part of natural history after surgical interventions [1]. This condition leads to a nonspecific systemic and often local inflammatory response syndrome without bacterial infection. Unspecific serum parameters used for diagnosis of infections, such as C-reactive protein (CRP) or white blood cell count (WBC), are frequently elevated and therefore differentiation between presence of early postoperative bacterial infection and harmless unspecific inflammatory syndrome remains difficult [2].

Procalcitonin (PCT) is widely used as a diagnostic marker of sepsis and systemic inflammatory response syndrome (SIRS) [3] and has been shown to be a more accurate marker in detection of early postoperative infection after cardiac, intestinal and major neural surgeries compared to the standard laboratory parameters (such as CRP and WBC [1, 4–6]). While in healthy persons PCT is mainly produced as a precursor hormone of calcitonin by the parafollicular cells of the thyroid, alternative pathological pathways in patients with inflammation and sepsis were described. In these cases inflammatory cytokines such as TNF-alpha, IL-1beta and fragments of cell walls or membranes of microbes like lipopolysaccharides or peptidoglycans may induce PCT production. Several investigators have reported a cut-off level of 0.5 micrograms/L indicating high probability for systemic bacterial infection [7–10].

However, to prevent unnecessary, painful, and time- and money-consuming revision surgery after primary total hip or knee replacement, a reliable serum marker indicating bacterial infection would be a wishful adjunct in the decision making process after surgery. For orthopaedic surgery only few data exists about usefulness of PCT for early diagnostics of bacterial infection. In chronic periprosthetic infection, PCT has been found to be a highly specific but not very sensitive surrogate marker and should be used to rule out false positive cases detected by elevated CRP and Interleukin 6 serum levels [11]. A recent evidence level 2 study demonstrated PCT to be a helpful diagnostic marker supporting clinical and microbiological findings for more reliable differentiation of infectious from noninfectious causes of fever after orthopaedic surgery [12]. Several investigations showed the postoperative course of PCT to vary depending upon the type of surgery and therefore no general assumption for orthopaedic interventions could be made [13, 14]. So far no data about the normal early postoperative PCT serum level course in uneventful primary hip prosthesis are available. The goal of the present study is to characterise the early postoperative baseline of serum PCT levels compared to CRP, WBC, and IL-6.

## 2. Material and Methods

The study was conducted in our Orthopaedic Department between June 2009 and April 2010, where 31 individuals (19 males and 12 females) with an age older than fifty years (52–81, mean 67) receiving a primary total hip replacement were prospectively included in our study group. Exclusion criteria were history of previous joint infection, acute or chronic systemic inflammatory disease, coagulopathy, and malignoma. All patients with preoperatively elevated CRP serum level above 5 mg/L were also excluded. The local ethics committee approved the study protocol and informed consent from all patients was obtained prior to surgery. Operational procedure comprised standardised unilateral minimal invasive implantation of an uncemented total hip prosthesis through an anterior approach between the tensor fascia lata and rectus femoris muscle.

Blood samples of all patients were taken one day before surgery and each day from the first to the fifth day postoperatively. All blood samples were taken every morning within a range of 3 hours and processed within two hours after harvesting. Serum levels of CRP (Hitachi P-Module, Roche Diagnostics Mannheim), WBC (ADVIA 2120, Siemens), PCT (KRYPTOR, B.R.A.H.M.S., Hennigsdorf, Germany), and IL-6 (Enzyme immunoassay, immulite 2500, Siemens healthcare Diagnostics AG), were measured at the Institute for Clinical Chemistry at University Hospital Zurich. Descriptive data analyses were carried out with a commercial statistical package (Analyse It for Microsoft Excel).

In seven patients only four days of postoperative blood sampling were available because of early return to domicile.

## 3. Results

**Figure 1:**
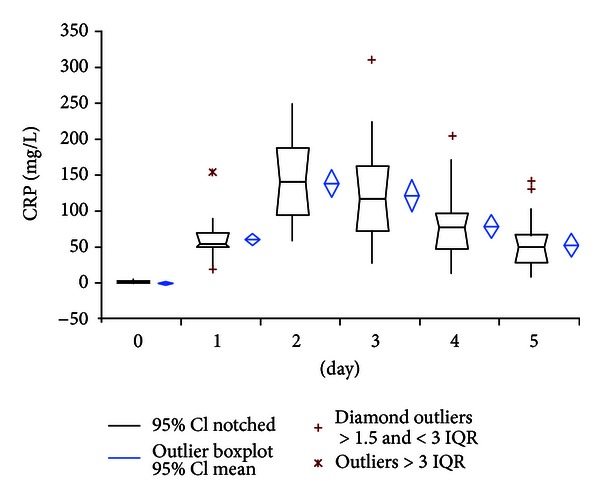
Course of CRP serum levels.

Preoperative serum levels of CRP (normal serum value: <5 mg/L) and WBC (normal serum value: 3–9.6 × 10^3^/*μ*L) were consistently low with an average of 1.88 mg/L (95% CI: 1.33–2.44) and 6.79 × 10^3^/*μ*L (95% CI: 6.27–7.31), respectively. The preoperative values of PCT (normal serum value: <0.1 *μ*g/L: no bacterial infection; >0.5 *μ*g/L: systemic bacterial infection) and IL-6 (normal serum value: <3.3 ng/L) showed also low levels in all patients with a mean of 0.05 *μ*g/L (95% CI: 0.04-0.05) and 2.23 ng/L (95% CI: −0.13–4.59), respectively. Postoperative CRP levels showed a widespread pattern of the individual courses on a relatively high level during the whole period of observation. Peak measurements of CRP occurred at postoperative day 2 in almost all patients. At the fifth and last day of measurements the mean value was still 53.31 mg/L (95% CI: 38.73–67.89) (Figure 1).

**Figure 2:**
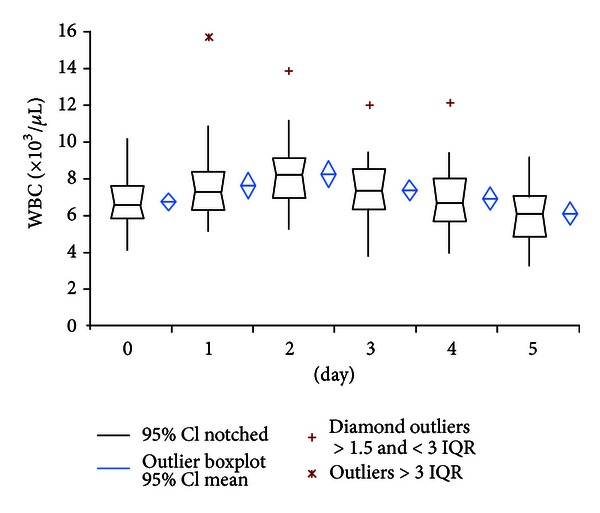
Course of WBC serum levels.

 WBC values showed also heterogenic distribution. A small peak of 8.31 × 10^3^/*μ*L (95% CI: 7.64–8.98) at day 2 was also seen in WBC measurements (Figure 2). 

**Figure 3:**
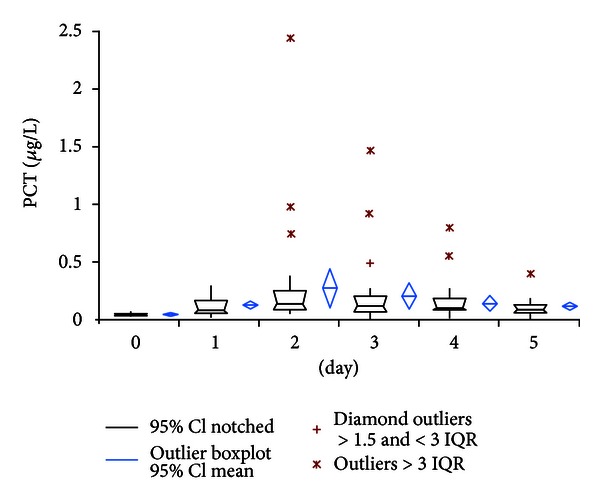
Course of procalcitonin serum levels.

PCT levels showed a more uniform course from day 1 to day 5 with peak values of 0.28 *μ*g/L (95% CI: 0.12–0.45) at the second postoperative day. At day 5 the mean value returned to 0.13 *μ*g/L (95% CI: 0.09–0.17). Two patients showed a high-level PCT course with PCT peak levels of 2.45 and 0.98 *μ*g/L, respectively, at day 2 (Figure 3). From the clinical point of view these findings cannot be explained; there were no clinical events in these patients neither during the hospital stay nor during the 3-month postoperative followup. Over the whole measurement period PCT mean value stayed below the 0.5 *μ*g/L cut-off level for systemic bacterial infection [7–10].

**Figure 4:**
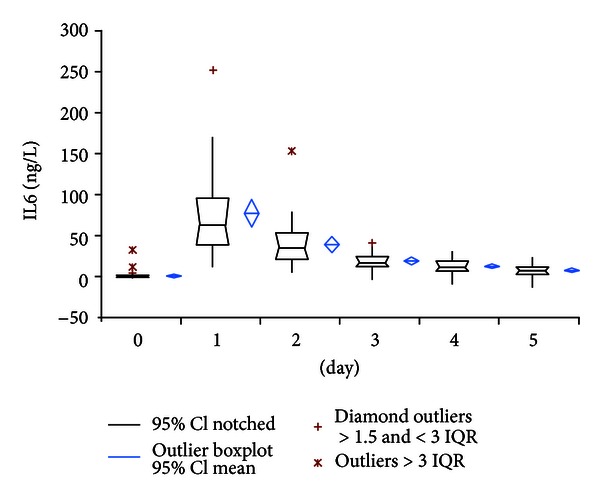
Course of Interleukin-6 serum levels.

In contrast to all other parameters IL-6 serum values showed initially high variance of the interindividual courses with peak levels of 77.19 ng/L (95% CI: 58.50–95.88) at the first postoperative day and rapid decline of values to almost preoperative levels at day 5 to 8.40 ng/L (95% CI: 6.01–10.79) (Figure 4).

Clinically, during the whole period of observation up to the 6-week followup no patient showed local or systemic complications, such as fever or wound healing problems. All patients had quick recovery time with early mobilisation and ambulation capacity within the hospital stay. Therefore in all patients clinically manifesting prosthetic infection could be excluded.

## 4. Discussion

Larsson et al. in 1992 reported about the superiority of CRP in detection of early postoperative infection after elective orthopaedic surgery compared to erythrocyte sedimentation rate (ESR). This is due to the fact that CRP values dropped postoperatively to normal levels (<10 mg/L) within 21 days compared to ESR level which needed 42 days to normalize after uncomplicated surgery [15]. In the past decade PCT was established as a surrogate marker for bacterial infection not only because of quicker metabolism than CRP but also for better specificity in detection of bacterial infection. Today PCT is a routine parameter in the diagnosis of SIRS and sepsis in many Intensive Care Units and is also increasingly used as monitoring and steering tool for antibiotic administration [3, 16]. For detection of early postoperative infection, several investigations of different surgical branches such as cardio-thoracic, abdominal, or neural surgery considered PCT to be a valuable marker and superior to CRP or WBC due to its nonresponding character in unspecific postoperative systemic inflammatory syndrome [1, 4–6]. Laifer et al. reported about PCT serum concentrations after uncomplicated major neurosurgery lower than 0.2 ng/mL compared to CRP with mean elevation of 14 mg/L (range 3–95 mg/L) in the early postoperative phase [5]. Jebali et al. showed PCT peak levels 4.4-fold higher in infected patients than in uneventful postoperative courses after cardiopulmonary bypass [4]. 

Today in orthopaedic surgery PCT is not widely used and only few studies on this topic are available. For chronic periprosthetic infection Bottner et al. reported high specificity (0.98) but low sensitivity (0.33) in detection of deep chronic periprosthetic infection with PCT levels >0.3 ng/mL while the combination of CRP and IL-6 levels >32 mg/L and 12 ng/L, respectively, showed high sensitivity (0.95). Therefore he recommended PCT measurement only to rule out false positive cases [11]. In recently published study, which focused on new-onset postoperative fever in 103 patients after orthopaedic surgery, PCT was named a highly significant predictor for postoperative infection [12].

The fact that surgical intervention leads to induction of PCT by upregulation of proinflammatory cytokines such as tumor necrosis factor-(TNF-) a, Interleukin-1beta, or IL-6 may explain slight initial increase of postoperative values in absence of bacterial infection [17–19]. It may also be the reason for different courses of postoperative PCT measurements varying with type of surgery as reported by Meisner et al. [13]. Since the amount of postoperative PCT increase depends on type of surgery, characteristics of postoperative PCT baselines cannot be generalised for all types of orthopaedic procedures.

However, similar to observations in cardiac, intestinal, or neural surgery, our results showed uniform sub-pathological PCT levels with a small and narrow peak at postoperative day 2 and rapid decrease to fifth postoperative day in uneventful primary hip replacement. In contrast, elevated CRP values persisted during the whole period of observation and showed broad scattered range of individual courses. WBC also showed a wide variety of courses but remained under the commonly used cut-off level of 10 × 10^3^/microL in contrast to reports of elevated WBC levels after cardiac or major neural surgery [4, 5]. Consistent with its quicker metabolism IL-6 showed a very rapid decline of values within two days postoperatively. Despite of quick return to preoperative values, the value of IL-6 for determination of bacterial infection remains critical because of low specificity as reported by Oberhoffer et al. [20].

Nevertheless our study has some limitations regarding the number of patients and lack of reference group with early prosthetic infection. The latter because of very low postoperative infection rate after primary total hip replacement in the clinic where the study was conducted (<1%). Thus the study design was declared as purely descriptive without any determinations of pathological cut-off values and only indirect information in terms of possible infectious course was accepted.

## 5. Conclusion

The present study clearly shows uniform pattern of postoperative courses of PCT and IL-6 with early elevation of serum levels and a quick return of values to almost preoperative levels within the first week after the intervention compared to WBC and CRP concentrations which remained on a high level and therefore both parameters may be a useful adjunct in detection of early postoperative infection after primary total hip replacement.
